# First Principles Calculations of Atomic and Electronic Structure of ${\mathrm{Ti}}_{\mathrm{Al}}^{3+}$- and ${\mathrm{Ti}}_{\mathrm{Al}}^{2+}$-Doped YAlO_3_

**DOI:** 10.3390/ma14195589

**Published:** 2021-09-26

**Authors:** Sergei Piskunov, Aleksejs Gopejenko, Vladimir Pankratov, Inta Isakoviča, Chong-Geng Ma, Mikhail G. Brik, Michal Piasecki, Anatoli I. Popov

**Affiliations:** 1 Institute of Solid State Physics, University of Latvia, Kengaraga Street 8, LV-1063 Riga, Latvia; agopejen@cfi.lu.lv (A.G.); vpank@cfi.lu.lv (V.P.); intai@cfi.lu.lv (I.I.); popov@cfi.lu.lv (A.I.P.); 2 College of Sciences & CQUPT-BUL Innovation Institute, Chongqing University of Posts and Telecommunications, Chongqing 400065, China; macg@cqupt.edu.cn (C.-G.M.); mikhail.brik@ut.ee (M.G.B.); 3 Institute of Physics, University of Tartu, W. Ostwald Str. 1, 50411 Tartu, Estonia; 4 Academy of Romanian Scientists, Ilfov Str. No. 3, 050044 Bucharest, Romania; 5 Faculty of Science and Technology, Jan Długosz University, Armii Krajowej 13/15, PL-42200 Częstochowa, Poland; m.piasecki@ujd.edu.pl; 6 Department of Inorganic Chemistry, Uzhhorod National University, 46 Pidhirna Str., 88000 Uzhhorod, Ukraine

**Keywords:** YAlO_3_, substitutional point defects, Ti-dopant, electronic structure, ab initio modelling

## Abstract

In this paper, the density functional theory accompanied with linear combination of atomic orbitals (LCAO) method is applied to study the atomic and electronic structure of the Ti^3+^ and Ti^2+^ ions substituted for the host Al atom in orthorhombic *Pbnm* bulk YAlO_3_ crystals. The disordered crystalline structure of YAlO_3_ was modelled in a large supercell containing 160 atoms, allowing simulation of a substitutional dopant with a concentration of about 3%. In the case of the Ti^2+^-doped YAlO_3_, compensated *F*-center (oxygen vacancy with two trapped electrons) is inserted close to the Ti to make the unit cell neutral. Changes of the interatomic distances and angles between the chemical bonds in the defect-containing lattices were analyzed and quantified. The positions of various defect levels in the host band gap were determined.

## 1. Introduction

It has long been well known that compounds of Y, Al and O form the following three crystal structures: Y_3_Al_5_O_12_ (YAG), Y_4_Al_2_O_9_ (YAM) and YAlO_3_ (YAP). There are numerous studies of their spectroscopic properties, in pure form and with different impurities. For example, Ce^3+^ spectra in YAG were studied recently in Refs. [1,2,3,4]. The same Ce^3+^ impurity in YAM was a subject of spectroscopic investigations in Refs. [5,6], whereas triply (Yb^3+^/Ho^3+^/Tm^3+^) doped YAM nanoparticles were investigated in Ref. [7].

Yttrium aluminum perovskite (YAP) plays an important role in materials science research as an excellent model system for in-depth studies of the defect formation and their influence on its optical properties, on the one hand, and development and improvement of the already existing applications, on the other. Such an interest is explained by two main factors. First of all, there exists the possibility of doping with many transition metal (TM) and rare earth (RE) ions, and not all crystals can offer such an opportunity. Various TM ions can occupy the Al site, whereas RE ions can be easily incorporated at the Y site. The second important circumstance is that YAP has a wide band gap, which in different publications was reported to range from 8.5 eV to 9.0 eV [8,9]. Many defects and/or impurity ion energy levels can be located within the forbidden gap, which will manifest themselves in the appearance of additional absorption/emission peaks in the defect-containing YAP spectra [9,10,11,12,13,14].

Historically, interest in YAP as an attractive material for doping with transition metal and rare earth ions has been driven due to its potential application [13,14] in solid-state lasers capable of operating at shorter wavelengths than classical Al_2_O_3_:Ti lasers [15]. It is expected that tunable laser crystal material, such as Ti-doped YAlO_3_, can demonstrate absorption range from 350 to 550 nm, as well as fluorescence range from 540 to 800 nm [16]. Because of these expectations, research on optical, luminescence and laser properties has been under the study of several research centers. Yamaga et al. [15] have performed detailed low temperature measurements of the zero-phonon lines of Ti^3+^ ions in YAP, YAG and Al_2_O_3_ at 10 K. The emission spectrum of Ti^3+^ in YAP consists of an intense zero-phonon line at 539.7 nm and with full width at half maximum (FWHM) of 30 cm^−1^ accompanied by a rather weak line at shorter wavelength 537.1 nm with FWHM of 55 cm^−1^. It was reported that the zero-phonon lines of Ti^3+^:YAP are strongly polarized perpendicular to the tetragonal axis and those of Ti^3+^:Al_2_O_3_ parallel to the trigonal axis. Detailed study of the donor and acceptor states and corresponding charge-transfer (CT) transitions in YAP:Ti^3+^/Ti^4+^ was done by Basun at al [9]. In particular, through the conductivity, optical absorption and luminescence measurements, it was found that the Ti^3+^ ground state is situated at about 4.60 ± 0.1 eV below the conduction band. Furthermore, a titanium-bound exciton band at about 3.60 eV above the Ti^3+^ ground state was identified. Another important result is that CT transition from the Ti^4+^ to Ti^3+^ ground state was found to be at 4.20 ± 0.1 eV. The results obtained suggest that the band gap energy *E_g_* of YAP is 8.8 ± 0.2 eV. This value is in good agreement with data of Lushchik et al. [8]; from there we can conclude that *E_g_* is in the range 8.5–9.0 eV. Two different processes, namely, Ti^3+^/Ti^4+^ pair formation and corresponding hole trapping at oxygen ions adjacent to a lattice defect are responsible for the broad parasitic photo-induced absorption [17].

A few examples that emphasize the diversity of applications of the doped YAP host are as follows: (i) scintillating applications (especially when doped with the La^3+^, Ce^3+^, Pr^3+^ ions) [18,19,20,21,22,23,24,25,26,27,28]; (ii) thermoluminescent material (YAP with Mn ions) [29,30,31,32,33,34]; (iii) solid-state laser applications [35,36] and (iv) electroluminescent devices [37]. Various aspects of the doped YAP crystal were studied theoretically, such as crystal field splittings of the Mn^3+^ and Mn^4+^ energy levels [38], splittings of the Ce^3+^ 4*f* and 5*d* states [39] and Ce^3+^ 4*f* ground state position in the YAP band gap [40,41]. The titanium impurities in YAP were studied experimentally in a number of references, for example, Ti^3+^ and Ti^4+^ excitation spectra [42,43,44,45], EPR spectra of Ti^3+^ [17,46] and charge transfer processes [9]. The trap defect levels in the YAP band gap were analyzed experimentally in Refs. [18,47]. The existing measurements of the structural optical, thermal and mechanical properties of pure and Ti-doped YAlO_3_ important for practical applications are summarized in Table 1.

As seen in the above given references, a vast majority of publications were focused on the Ti^3+^ or Ti^4+^ ions. However, if the Ti^3+^ ions capture an electron, another type of impurity center, Ti^2+^, will be formed. Such a center in YAP is less studied, and the present paper offers a theoretical insight into its properties.

One of the key factors that plays a crucial role in assessing perspectives of a given material for its optical applications is the location of the impurity ion ground state in the host band gap. In view of this, in the present paper we continue our earlier studies of the defects electronic structure in the YAP crystal [34,38,39,48,49] by performing the first principles calculations for the pristine YAP, Ti^3+^ and Ti^2+^ doped YAP as well as for the *F* color center (the oxygen vacancy with two electrons) in the YAP lattice. All calculations have been performed within the formalism of hybrid density functional theory (DFT). Comparison of the calculated results for the defect-containing YAP with those for the pristine material allowed us to establish the influence of defects on the structural and electronic properties of the studied host material.

**Table 1:** Structural and optical properties of orthorhombic YAP and YAP:Ti.

a_0_, b_0_, c_0_ (Å)	5.179, 5.329, 7.370	[50]
Y sites/cm^3^	1.97 × 10^22^	
Bulk modulus, B (GPa)	192	[50]
Hardness (Knoop value)	1310	[13]
Melting point (°C)	1875	[13]
Thermal conductivity (W/cm-°C at 25°C)	0.11	[13]
Thermal expansion coefficient (10^−6^/°C, at 25°C)	2.2	[13]
Optical transmission (absorption coefficient < 1.0 cm^−1^)	0.29–5.9 μ	[13]
Refractive index (6328 Å) (a = z, b = y, c = x)	*n_a_* = 1.97 *n_b_* = 1.96 *n_c_* = 1.94	[13]
Band gap energy, eV	8.5–9.0 8.8 ± 0.2	[8] [9]
Ti-ion related optical absorption bands (eV)	7.08 (Ti^3+^-related) 5.76 (Ti^4+^-related) 5.27 (Ti^4+^-related) 5.39 (Ti^3+^ → Ti^4+^) 4.42 (Ti^3+^-related) 4.42 (^2^T_2g_ → 4S, Ti^3+^) 4.20 (Ti^4+^ → Ti^3+^) 2.88 (^2^T_2g_ → ^2^E, Ti^3+^) 2.87 (Ti^3+^) 2.81 (Ti^3+^) 2.53 (Ti^3+^) 2.52 (Ti^3+^) 2.50 (^2^T_2g_ → ^2^E, Ti^3+^)	[9] [9] [9] [16] [9] [43] [9] [43] [16] [9] [9] [16] [43]
Ti-ion luminescence band	3.02 (Ti^4+^) 2.10 (Ti^3+^, d-d transition) 2.06 (Ti^3+^, d-d transition) 1.99 eV (Ti^3+^, d-d transition)	[9] [43] [9] [16]
*F* center: optical absorption (eV) luminescence (eV)	5.84 and 5.15 2.95	[51] [51]
*F*^+^ center: optical absorption (eV)luminescence (eV)	6.5, 5.63, and 4.3 3.49	[51] [51]

The paper is organized as follows: Section 2 contains all relevant details of the YAP structure and calculating settings, Section 3 describes all calculated results in relation to the experimental data and (when available) calculated values, and, finally, Section 4 concludes the paper with a short summary.

## 2. Computational Details

The DFT approach is based on the Hartree-Fock method using the HSE06 Hamiltonian [52,53] as implemented in total energy CRYSTAL17 computer code [54], which was used in all calculations. The computer simulation began from complete geometry optimization to be sure our modelling was able to reproduce the basic crystal parameters, e.g., lattice constant and optical band gap of ideal YAP. Then, the influence of the presence of the Ti^3+^ dopant and the Ti^2+^ dopant and its compensating *F*-center on YAP atomic and electronic structures were studied using a supercell (SC) approach. The localized Gaussian-type functions (GTFs) in the form of basis set (BS) centered on atomic nuclei for expansion of crystalline orbitals as linear combinations of atomic orbitals (CO LCAO) were employed. The hybrid exchange–correlation functional HSE06 has been used according to direction given in Refs. [52,53,54]. The Triple-Zeta Valence with Polarization quality BSs for titanium, alumina and oxygen were taken from Ref. [55], while for yttrium the Hay-Wadt effective core pseudopotential BS was taken directly from Ref. [54]. Use of LCAO GTF approach greatly facilitates the calculation of effective charges and bond population from the wave functions using the Mulliken population scheme [56,57].

**Figure 1:**
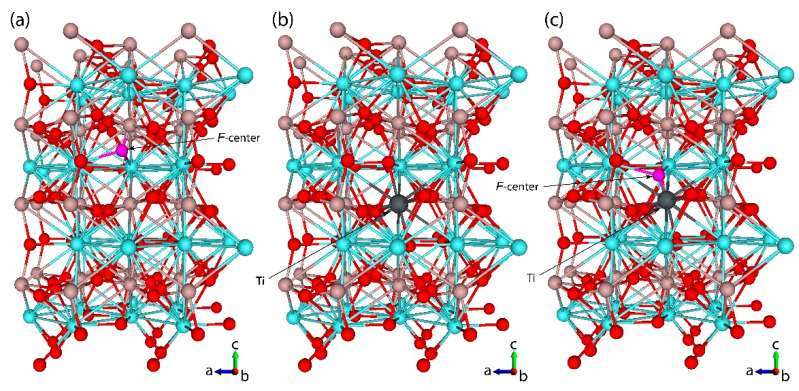
Equilibrium structure of 2 × 2 × 2 supercell of orthorhombic Pbnm YAlO_3_ with (**a**) F-center, (**b**) Ti^3+^ ion and (**c**) Ti^2+^ ion substituted for host Al^3+^ atom. The supercell contains 160 atoms in a periodically repeated unit. The Ti dopant is shown as a dark-grey ball, the compensating *F*-center is a small pink ball, and the Y, Al, and O sublattices are shown in light blue, brown, and red, respectively. The “b” crystallographic axis is perpendicular to the figure plane and is directed outward.

YAP possesses a perovskite structure of the GdFeO_3_ type [58]. Its orthorhombic unit cell (Pbnm space group) contains four formula units with two non-equivalent oxygen positions. In order to simulate the isolated substitution defect, the orthorhombic unit cell was extended to a 2 × 2 × 2 SC. The SC retains the orthorhombic symmetry and contains 160 atoms with the distance between periodically repeated defects of 10.38 Å [48]. To model the F-center, we employed “ghost” BS [53], meaning the atomic orbitals of removed oxygen are left behind. This technique facilitates the accurate description of the electron distribution within the vacancy and allows us to mimic the *F*^+^-center, which is a compensating defect to simulate a Ti^2+^ substitutional dopant. The equilibrium geometry is obtained using an analytical optimization method as implemented in the CRYSTAL code [54]. To provide a balanced summation in both direct and reciprocal lattices, the reciprocal space integration has been performed by sampling the SCs Brillouin zone with a 3 × 3 × 3 Pack-Monkhorst mesh [59] that gives 14 k-points in total. The calculations have been considered as converged only when the total energy differs by less than 10^−8^ a.u. in two successive cycles of the SCF (Self-Consistent Field) procedure. Within the self-consistency, the accuracies (tolerances) of 10^−8^ were chosen for calculations of Coulomb and exchange integrals [54]. The visualizing software VESTA (https://jp-minerals.org/vesta/en/, accessed on 25 September 2021) was used to present equilibrium YAP structures in Figure 1.

## 3. Results and Discussion

### 3.1. Structural Properties

Figure 1 shows the considered supercells for each of the above-described structural models. Influence of the structural defects (substitutional atoms and vacancies) on the YAP lattice geometry is considered by comparing the optimized interatomic distances and angles between chemical bonds for all above-described structural models.

Table 2 lists the lattice constants and atomic coordinates obtained in this study, as well as their experimental values [50], calculated using the HSE06 exchange-correlation function within DFT [60]. It can be easily seen that the equilibrium lattice constants (a_0_ = 5.189 Å, b_0_ = 5.317 Å, and c_0_ = 7.388 Å) calculated in this study are in very good agreement with both those experimentally measured (a_0_ = 5.179 Å, b_0_ = 5.329 Å, and c_0_ = 7.370 Å [50]) and those previously calculated (a_0_ = 5.179 Å, b_0_ = 5.342 Å, and c_0_ = 7.367 Å [60]). In addition, the experimental and calculated fractional coordinates of all ions in a unit cell expressed in the crystal lattice constants’ units are also very close to one another. Thus, we conclude that the chosen computational approach allows us to obtain reliable data for the Ti-doped YAP crystal.

The nearest environment of the Al^3+^ ions in YAlO_3_ is a deformed octahedron made of six oxygens. The second coordination sphere around Al^3+^ ions contains six Y^3+^ ions. The oxygen ions laying on the opposite sides of the central Al^3+^ ion are located on the same straight line with the central ion, making the angles of 180 degrees—this is an important observation, which will be used to illustrate the difference with the defect-containing YAlO_3_ lattice.

Isovalent substitution of the Al^3+^ ion for the Ti^3+^ ion is accompanied by the expansion of the TiO_6_ octahedron, which is in line with a greater Ti^3+^ ionic radius in six-fold coordination (0.67 Å, if compared with 0.535 Å for Al^3+^ [61]. All O–Ti–O angles inside the TiO_6_ octahedron are not changed, which is anticipated since the electric charges of the substituting and substituted ions are the same. Therefore, such an isovalent doping leads only to an expansion of the crystal lattice while keeping all angles between the chemical bonds the same, as in the neat lattice.

**Figure 2:**
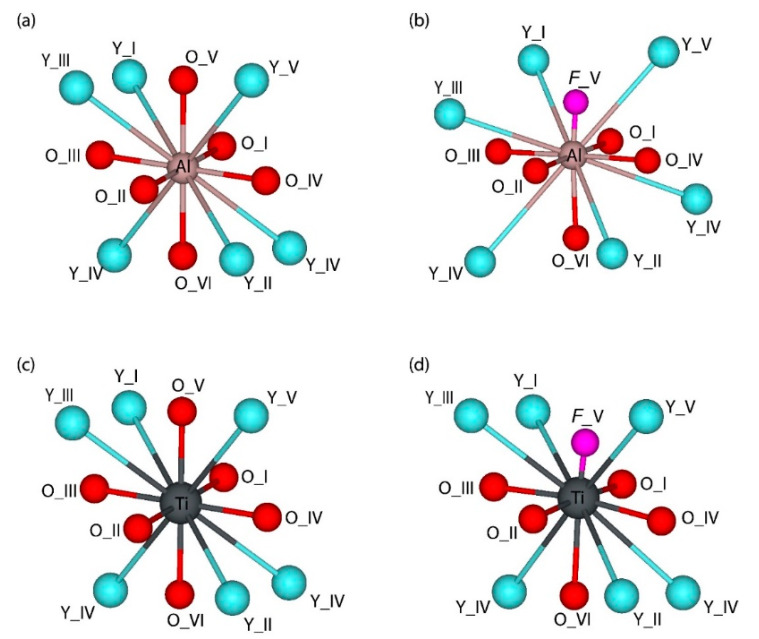
Equilibrium positions of oxygen octahedra in the 2 × 2 × 2 supercell structure of orthorhombic *Pbnm* YAlO_3_. (**a**) pristine YAlO_3_, (**b**) YAlO_3_ with *F*-center, (**c**) YAlO_3_ containing Ti^3+^ ion and (**d**) YAlO_3_ containing Ti^2+^ ion substituted for host Al^3+^ ion. See Table 3 for bond lengths and angles.

Presence of the oxygen vacancy or substitution of the Al^3+^ by the Ti^2+^ ion considerably lowers the symmetry of the AlO_6_ or TiO_6_ octahedron because the oxygen ions located on opposite sides of the central ion are no longer on the same straight line, deviating from the 180° angles (Table 3). In the long run, such symmetry lowering may lead to the enhancement of the emission lines of impurity ions located at those sites. It should be noted that the presence of the oxygen vacancy considerably shortens the distance to the nearest Al (or Ti) ion (Figure 2 and Table 3).

### 3.2. Electronic Properties

In the ternary compounds, such as YAP, two pairs of bonding atoms can be selected, like Y–O and Al–O in this particular case. Their chemical properties are not identical, as they depend on the atomic numbers, charges, electron configurations and electronegativities of the ions involved into these bond formations. The peculiar bonding features can be analyzed by considering the effective Mulliken charges and bond orders as follows. The calculated Mulliken charges (Table 4) deviate from those prescribed by the formal valences derived from the chemical formula (+3 for Y and Al, −2 for O), which is an indication of the covalent bonds between the ions in YAlO_3_ crystal lattice. The charge of the yttrium ions is closer to the “+3” value than that of the aluminium ions; therefore, the Y–O ions are more ionic, whereas the Al–O ions are more covalent. The oxygen ions effective charges for a pristine YAlO_3_ also follow the geometrical structure: the charges of the ions located at the same distance from the central Al^3+^ ion are the same. Moreover, the absolute values of the ions located further from the central ion are slightly greater than those ones of the closer ions. This can be explained by weaker overlap of the oxygen and aluminium wave functions and increased degree of ionicity of the corresponding Al–O bonds. *F*-centers in pristine and Ti^2+^-doped YAP attract 0.53 e and 0.66 e, correspondingly.

The atomic orbitals of the constituting atoms in the YAP lattice are hybridized because of chemical bond formation. Due to these effects, the oxygen 2*p* ions produce a minor contribution to the conduction band, whereas the cation’s unfilled states can be traced down by their small percentage in the valence band. These effects are different in the Y–O and Al–O pairs and are represented in detail in Table 4, which lists the results of the Mulliken effective charge and bond order calculations.

**Figure 3:**
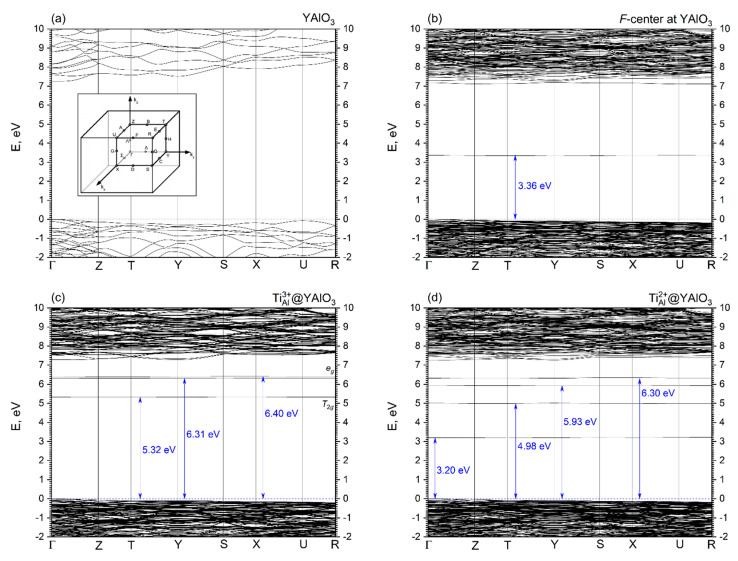
Electronic band structure as calculated for (**a**) perfect orthorhombic YAlO_3_ bulk crystal, (**b**) *F*-center and YAlO_3_, (**c**) Al-site Ti^3+^-doped YAlO_3_ and (**d**) Al-site Ti^2+^-doped YAlO_3_. Zero at the energy scale corresponds to the top of the valence band. Inset shows schematically the Brillouin zone and high symmetry points of Pbnm space group.

**Figure 4:**
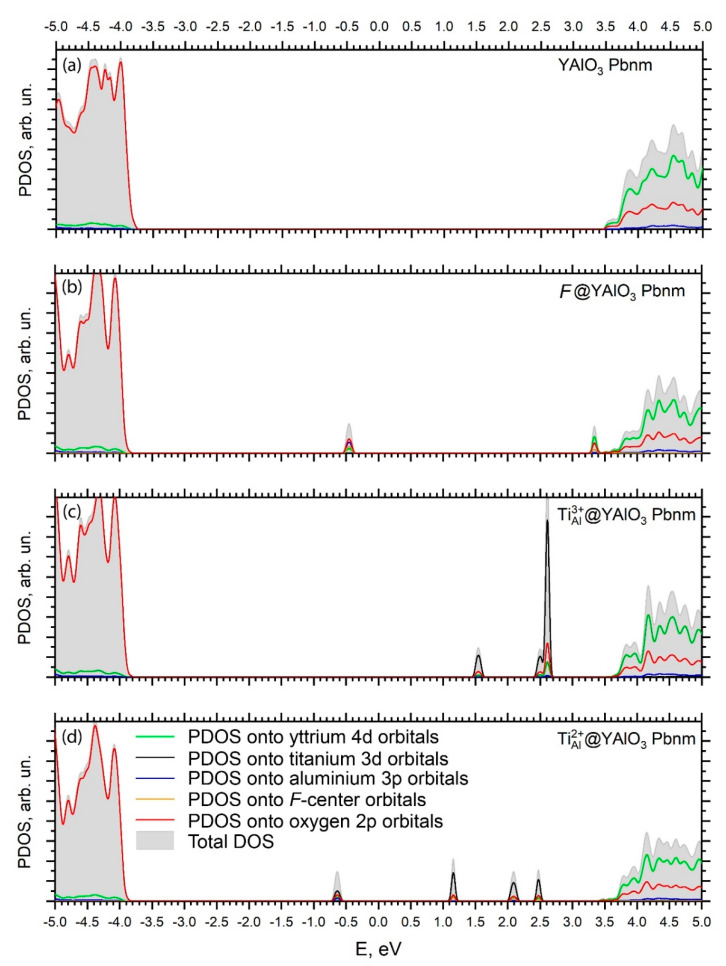
Equilibrium positions of oxygen octahedra in the 2 × 2 × 2 supercell structure of orthorhombic *Pbnm* YAlO_3_. (**a**) pristine YAlO_3_, (**b**) YAlO_3_ with *F*-center, (**c**) YAlO_3_ containing Ti^3+^ ion and (**d**) YAlO_3_ containing Ti^2+^ ion substituted for host Al^3+^ ion. See Table 3 for bond lengths and angles.

Figure 3 and Figure 4 show band structures and density of states (DOS) calculated for pristine, F-center containing, Ti^3+^- and Ti^2+^-YAP. The direct Γ-Γ band gap calculated in this study for pristine YAP has a width of 7.26 eV [48] and is in good agreement with the experimentally detected band gap of 8.5 eV [8]. The band structures calculated for all four perovskite structures under study look similar and agree with band structures published previously in the literature using different ab initio methods [60]. Position of the bottom of the conduction band (CB) practically does not change with respect to the top of the valence band (VB), while the levels induced by defects are present within the YAP’s optical gap. The VB top and CB bottom of pristine YAP consist of the O 2*p* and Y 4*d* states with an admixture of the Al 3*p* orbitals, respectively.

In the case of *F*-center (Figure 3b and Figure 4b), an occupied level is located at 3.36 eV above of the VB top. This level has a very small dispersion of 25 meV, meaning the *F*-center can be treated as an isolated defect within the framework of the chosen model. The defect level induced by *F*-center consist mainly of admixture of *F*-center orbitals with the O 2*p* and Al 3*p* orbitals (Figure 4b) that is in line with relatively short Al–*F*-center bond of 1.22 Å (Table 3). An empty level also induced by *F*-center in pristine YAP is located very close to its bottom of the CB (Figure 4b) and contains also an admixture of the Y 4*d* states. In fact, this empty level may form the bottom of the CB, making YAP to act as an indirect Γ-Z semiconductor.

The Ti^3+^ ion substituting the host Al ion in the YAP lattice induces three defect levels inside its optical gap (Figure 4c). Due to distortion of oxygen octahedra around the Ti dopant, the *e_g_*-*t_2g_* splitting of the Ti 3*d* orbitals takes place. The occupied *t_2g_* level is located 5.32 eV above the VB top and has the dispersion (energy interval, in which this level is localized) of 20 meV. This level consists mainly of the Ti 3*d* states with a small admixture of the O 2*p* states. The other two empty levels are located close to each other at 6.31 and 6.40 eV above the top of the VB, respectively. Their dispersions are 5 and 10 meV, respectively. Empty levels induced by the Ti^3+^ dopant consist of the Ti 3*d* states with a small admixture of the O 2*p* and Al 3*p* orbitals (Figure 3c).

YAP doped with the Ti^2+^ ions contains a compensating *F*-center to force a unit cell to be neutral. Presence of the *F*-center induces an occupied level in the YAP crystal at 3.20 eV above its VB top (Figure 3d). This level has a dispersion of 25 meV and consists of an admixture of *F*-center states, O 2*p* and Ti 3*d* orbitals (Figure 4d). The other three levels induced by the Ti^2+^ dopant are located at 4.98, 5.93, and 6.30 eV above the VB top, respectively (Figure 3d). The level at 4.98 eV is occupied, while two others are vacant. These three levels’ dispersions are 27, 7, and 38 meV, respectively.

Presence of defects, which affects the effective charges of the surrounding ions, also modifies slightly the VB profile, as is shown by Figure 4.

We note that the YAP host cannot be directly doped with the Ti^2+^. However, formation of Ti^2+^–*F*-center complexes is expected in YAP during, e.g., hard irradiation. Formation of titanium ions in this oxidation state is physically unavoidable for at least two reasons: (i) the perovskite crystals always contain oxygen vacancies. Removal of the negative charge would imply removal of the positive charge as well, hence Ti^2+^ can appear instead of Ti^3+^; (ii) the charge transfer transitions from the oxygen ions to the Ti^3+^ ions would lead to the formation of the Ti^2+^. According to the Mulliken population analysis performed in this study (Table 4), we predict the formation of Ti^2+^ ion with Mulliken effective charge of 1.07 *e* if the *F*-center is created in the Ti-doped YAP crystal. The Mulliken effective charge calculated for Ti^3+^-doped YAP if no *F*-center is present is 1.56 *e* (Table 4). Fermi level of Ti^3+^-doped YAP is positioned 5.31 eV above the top of the VB of the undoped crystal, while in case of possible formation of Ti^2+^–*F*-center complex the Fermi level is located 4.94 eV above of the VB top of pristine YAP or 0.37 eV below the Fermi level of Ti^3+^-doped crystal, assuming the coexistence of the Ti^3+^/Ti^2+^ dopants in YAP under, e.g., ionizing irradiation conditions when formation of oxygen vacancies (*F*-centers) is highly expected. In addition, we note that the formation of the Ti^2+^-dopant in YAP without the presence of the *F*-center is hardly possible and thus is beyond the scope of this study.

Finally, we must emphasize that understanding where the impurity levels are located in the band gap, how these levels change their position with a change in the charge state of the impurity ion and how they are located in relation to each other, as well as in relation to point defects (*F*-centers)—all this is very important for an accurate and detailed description of the processes of photo- and thermally stimulated conversion of point defects in wide-gap halides, oxides and perovskites [62,63,64,65,66,67,68,69,70,71,72,73,74,75,76].

## 4. Conclusions

Detailed calculations of the structural and electronic properties of the pristine defect-containing YAlO_3_ were performed in the present paper. Among the considered defects were *F-*center (oxygen vacancy), Ti^3+^ and Ti^2+^ ions, introduced instead of the Al^3+^ ion. Optimization of the crystal structures for each considered case has led to the following conclusions:

(i) Isovalent replacement of the Al^3+^ ion by the Ti^3+^ ion keeps the symmetry of the substitutional site; the only effect is a slight increase of the Ti–O distances as compared to the Al–O ones because of the difference in ionic radii of the Al^3+^ and Ti^3+^ ions. Our calculated position of the Ti^3+^ ground state in the YAP band gap (5.32 eV above the valence band top) agrees nicely with the value of about 5.21 eV derived by Rogers and Dorenbos in Ref. [77].
(ii) Appearance of the oxygen vacancy or replacement of the Al^3+^ ion by the Ti^2+^ ion with simultaneous formation of the charge compensating defects lowers the symmetry of the substitutional site by modifying the angles between the chemical bonds in the AlO_6_ or TiO_6_ octahedra.
(iii) Creation of the defects in the pristine YAlO_3_ structure leads to the formation of localized dispersionless defects’ energy levels in the host band gap. These levels are located in the central region of the band gap and closer to the CB bottom; they can significantly modify the host’s optical properties by causing additional defect-related absorption peaks in the optical spectra.

We note that appearance of the substitutional defects and F-centers in the ideal YAP crystal structure will be accompanied by (i) emergence of local vibrational modes and (ii) splitting of the host’s degenerated vibrational modes due to the symmetry lowering around these point defects, which is currently out of scope of the present paper.

## Figures and Tables

**Table 2:** Structural characteristics of YAlO_3_ crystal in orthorhombically distorted *Pbnm* perovskite structure.

Lattice Constants and Volume	Experiment, Ref. [50]			Calc. (HSE06), Ref [60]			This Study		
a_0_, Å	5.179			5.179			5.189		
b_0_, Å	5.329			5.342			5.317		
c_0_, Å	7.370			7.367			7.388		
V, Å^3^	203.49			203.82			203.86		
**Fractional Coordinates (In Units of the Lattice Constants)**									
**Atoms:**	**x**	**y**	**z**	**x**	**y**	**z**	**x**	**y**	**z**
Y	−0.012	0.053	0.25	−0.012	0.055	0.25	−0.011	0.050	0.25
Al	0	0.5	0	0	0.5	0	0	0.5	0
O1	0.084	0.477	0.25	0.084	0.478	0.25	0.082	0.479	0.25
O2	0.705	0.295	0.044	0.706	0.294	0.042	0.708	0.292	0.044

**Table 3:** Atomic structure of pristine and Ti-doped YAlO_3_ crystals in orthorhombically distorted perovskite structure. Bond lengths l are given in Å and bond angles α are in degrees. For atomic notations please see Figure 2.

	YAlO_3_	YAlO_3_:F	YAlO_3_:Ti^3+^	YAlO_3_:Ti^2+^
l_Al,Ti-O_I_	1.92	1.92	2.01	1.94
l_Al,Ti-O_II_	1.92	1.92	2.01	1.96
l_Al,Ti-O_III_	1.90	1.90	1.99	2.00
l_Al,Ti-O_IV_	1.90	1.90	1.99	2.00
l_Al,Ti-O_V,F_	1.90	1.22	1.97	1.21
l_Al,Ti-O_VI_	1.90	1.92	1.97	2.08
l_Al,Ti-Y_I_	3.02	2.98	3.02	2.98
l_Al,Ti-Y_II_	3.02	3.04	3.02	3.10
l_Al,Ti-Y_III_	3.15	3.12	3.17	3.15
l_Al,Ti-Y_IV_	3.15	3.16	3.17	3.25
l_Al,Ti-Y_V_	3.24	3.17	3.30	3.14
l_Al,Ti-Y_VI_	3.24	3.26	3.30	3.33
α_0_I-Al,Ti-O_II_	180.0	178.6	180.0	175.4
α_0_III-Al,Ti-O_IV_	180.0	178.5	180.0	172.8
α_0_V,F-Al,Ti-O_VI_	180.0	174.4	180.0	174.7
α_Y_I-Al,Ti-Y_II_	180.0	178.9	180.0	175.4
α_Y_III-Al,Ti-Y_IV_	180.0	179.0	180.0	175.8
α_Y_V-Al,Ti-Y_VI_	180.0	179.4	180.0	176.8

**Table 4:** Mulliken population analysis of pristine and Ti-doped YAlO_3_ crystals in orthorhombically distorted perovskite structure. Q is Mulliken effective charge in e, bond populations P are in e. Negative bond population means electronic repulsion. For atomic notations please see Figure 2.

	YAlO_3_	YAlO_3_:F	YAlO_3_:Ti^3+^	YAlO_3_:Ti^2+^
Q_Y_I_	2.46	2.42	2.45	2.40
Q_Y_II_	2.46	2.46	2.45	2.46
Q_Y_III_	2.46	2.45	2.45	2.45
Q_Y_IV_	2.46	2.46	2.45	2.46
Q_Y_V_	2.46	2.45	2.46	2.43
Q_Y_VI_	2.46	2.46	2.46	2.46
Q_Al,Ti_	1.77	1.26	1.56	1.07
Q_O_I_	−1.42	−1.44	−1.39	−1.35
Q_O_II_	−1.42	−1.43	−1.39	−1.37
Q_O_III_	−1.40	−1.43	−1.37	−1.38
Q_O_IV_	−1.40	−1.44	−1.37	−1.39
Q_O_V,F_	−1.40	−0.53	−1.35	−0.66
Q_O_VI_	−1.40	−1.41	−1.35	−1.38
P_Al,Ti-O_I_	0.28	0.38	0.16	0.11
P_Al,Ti-O_II_	0.28	0.37	0.16	0.15
P_Al,Ti-O_III_	0.27	0.34	0.15	0.05
P_Al,Ti-O_IV_	0.27	0.35	0.15	0.09
P_Al,Ti-O_V,F_	0.27	−0.62	0.16	−1.44
P_Al,Ti-O_VI_	0.27	0.18	0.16	−0.24

## Data Availability

The raw/processed data required to reproduce these findings cannot be shared at this time as the data also form a part of an ongoing study.
